# Inferring Static Hand Poses from a Low-Cost Non-Intrusive sEMG Sensor

**DOI:** 10.3390/s19020371

**Published:** 2019-01-17

**Authors:** Nadia Nasri, Sergio Orts-Escolano, Francisco Gomez-Donoso, Miguel Cazorla

**Affiliations:** University Institute for Computer Research, University of Alicante, P.O. Box 99, 03080 Alicante, Spain; sorts@ua.es (S.O.-E.); fgomez@dccia.ua.es (F.G.-D.); miguel.cazorla@ua.es (M.C.)

**Keywords:** surface electromyography sensor, dataset, gated recurrent units, gesture recognition

## Abstract

Every year, a significant number of people lose a body part in an accident, through sickness or in high-risk manual jobs. Several studies and research works have tried to reduce the constraints and risks in their lives through the use of technology. This work proposes a learning-based approach that performs gesture recognition using a surface electromyography-based device, the Myo Armband released by Thalmic Labs, which is a commercial device and has eight non-intrusive low-cost sensors. With 35 able-bodied subjects, and using the Myo Armband device, which is able to record data at about 200 MHz, we collected a dataset that includes six dissimilar hand gestures. We used a gated recurrent unit network to train a system that, as input, takes raw signals extracted from the surface electromyography sensors. The proposed approach obtained a 99.90% training accuracy and 99.75% validation accuracy. We also evaluated the proposed system on a test set (new subjects) obtaining an accuracy of 77.85%. In addition, we showed the test prediction results for each gesture separately and analyzed which gestures for the Myo armband with our suggested network can be difficult to distinguish accurately. Moreover, we studied for first time the gated recurrent unit network capability in gesture recognition approaches. Finally, we integrated our method in a system that is able to classify live hand gestures.

## 1. Introduction

The importance of technology and science in improving the quality of human health and facilitating human life has been amply demonstrated [1,2]. According to information from the LN-4 project (https://ln-4.org/), the amputee population can be estimated at 1.5 per 1000 persons and 30% of these are arm amputees [3]. In this case, using an upper-limb prosthesis is a popular solution. The main objective of this work is a system for amputees that can also be used as a robot remote control.

Most of the deployed cosmetic prosthetics resemble an arm or hand simply to offset the space left by the amputated limb. Fortunately, with constantly improving technology, efforts are focused on creating several variants of robotic arms and hands that could replace the lost limb with similar characteristics to a real hand in both appearance and movements. The prostheses available need complicated, cumbersome, expensive equipment, reducing demand.

However, the methods for controlling robotic prosthetics are a topic of interest in the literature. In addition, most of the control methods require surgery to be implanted for good prosthetic performance, and are intended to be permanent. However, technology advances, modifying and upgrading these mechanisms may be problematic, involving discomfort and significant extra costs. Amputees’ health may be endangered and the upgrade capabilities may be technically and economically limited.

There also exist control methods which are non-intrusive and benefit from surface electromyography (sEMG). A surface EMG is a useful non-intrusive technique for recording the electrical activity produced by muscles through surface sensors placed on parts of the body while muscles in that area are active. This technology is currently emerging as a substitute for the above-mentioned intrusive methods to control prosthetic limbs, with a focus on safety precautions against unknown consequences [4,5,6].

The most recent methods for controlling prosthetics leverage Machine Learning techniques. These classifiers receive labeled data from EMG sensors to learn and recognize the high level features of each pose. Subsequently, they take the output of the EMG sensors in real time shortly after the hand pose, and infer the signals and the features of the pose the user is trying to perform. The pose configuration is then sent to the motors placed in the prosthetic to move and replicate the intended pose.

An issue considered in this research is, the need of a large number of custom EMG sensors in some successful previous studies, and, therefore, their high cost [7,8]. This creates problems for users to access these methods, hence, in the present work, we attempted to create the maximum benefit at the minimum cost. Our proposed framework utilizes the Myo Gesture Control Armband, which is made of low-cost non-intrusive sEMG sensors (existing consumer product released by Thalmic Labs). In addition, this paper aims to expand the research to create a new dataset for six static hand gestures and use Gated Recurrent Unit (GRU) architecture for gesture recognition, which is new approach for this type of architecture.

Human–Robot Interaction (HRI) has been a noteworthy topic of academic research since the 20th century [9]. HRI research spans a wide range of fields dedicated to understanding, designing, and evaluating robotic systems and their services to humanity. There are several methods of human–robot interaction, each of them with their own benefits and deficiencies [10,11].

The main contributions of this paper are:

- The creation of a public dataset containing 35 subjects with six dissimilar hand gestures.
- A method for discrete hand pose classification with sEMG signals. The proposed method is based on deep learning and it obtains a high recognition accuracy.

The method presented in this paper can be used as a teleoperation method that an operator can remote and control a robot meticulously through six static hand gestures with any degree of freedom (DOF) in shoulder joint and elbow (shoulder joint includes three DOF and elbow includes two DOF). Teleoperation methods are useful in several fields, especially in some working environments that are dangerous and not suitable for operators. Several teleoperation methods were proposed [12,13] and there are some works with use of the Myo armband for the purpose of controlling robots with different methods [14,15,16].

The rest of the paper is organized as follows: Section 2 reviews the state of the art related to existing hand pose datasets, recorded using EMG and sEMG sensors. Moreover, we review existing learning-based approaches/systems that perform hand gesture and pose estimation using sEMG sensors. Next, in Section 3, the details of the capture device and sensor types that we used for composing the dataset are described and relevant details of the process of acquiring datasets are explained. Section 4 describes the system and architecture of the neural network used to train the hand gestures recognition dataset to be used for other hand poses to estimate the success of the system based on the proposals. Section 5 shows the obtained results and describes the experimental process. Section 6 presents the main conclusions of the present work and suggests some objectives for future works.

## 2. Background and Related Work

Hand gesture recognition via surface (sEMG) sensors placed on the arm has been a subject of considerable research with different features for applications and prosthetics. Quality of life for amputees is highly deficient in comparison to pre amputation (and losing their limb) but can be ameliorated with real-time control systems based on hand movements [17,18].

Nowadays, high-density surface electromyography (HD-sEMG) is of great importance in the medical community [19,20], and has also been used to recognize hand gestures and control muscle–computer interfaces (MCIs) [21,22,23]. A large number of electrodes are essential [24], although other methods exist which improve recognition accuracies using multi-channel sEMG and HD- sEMG electrodes [25,26].

**Figure 1:**
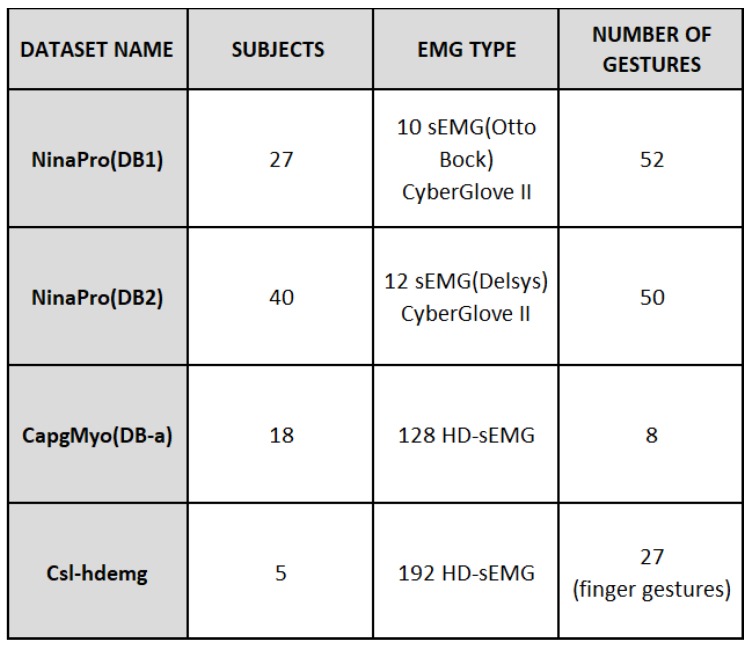
Public database summary table.

There are published datasets (See Figure 1) from several studies with a similar aim such as NinaPro (Non Invasive Adaptive Hand Prosthetics) [27], CapgMyo [28] and csl-hdemg [24]. In CapMyo and csl-hdemg, datasets were recorded by a large number of HD-sEMG with a high sampling rate using dense arrays of individual electrodes, in order to obtain information from the muscles [28]. Recently, a significant number of methods have been used which depend on HD-sEMG with 2D electrode arrays for gesture recognition approaches [22,23,29]. The capacity of HD-sEMG is currently the subject of research. Bearing in mind the existence of significant differences between sEMG signal and HD-sEMG results [29], we decided to work with sEMG.

Datasets which are acquired by HD-sEMG signals are not appropriate under our framework in this research and only NinaPro databases contain regular sEMG electrodes in their recording process. The NinaPro database was recorded from a sample of 78 participants (67 able-bodied persons, 11 trans-radial amputated persons, in three different phases) and 52 different gestures. In this project, 10–12 electrodes (Otto Bock 13-E200 (Ontario, ON, Canada), Delsys Trigno (Natick, MA, USA)) were placed on forearms, plus one CyberGlove II with 22 sensors to capture 3D hand poses. Four classification methods were applied on a five-signal feature. Because of the multiplicity of costly sensors and the various signal processing steps applied on raw sEMG data used in this research, we decided to work with raw signals. The NinaPro dataset does not fit the objective of this work [8]. In the continuation of the NinaPro research and development of their benchmark, a public dataset DB5 was recorded using a double Myo armband and a CyberGlove in order to reduce the expenses of the research with qualified results [30].

An investigation which focused on the evaluation between different machine learning methods exerted on EMG signals [31] provided interesting results and many studies highlight the success of deep learning techniques, especially ConvNet, in hand gesture classification [16,25,32]. They also demonstrate that using ConvNet as a feature extractor from sEMG images (or spectrograms) can achieve qualified accuracy [16,28,32] even with semi-supervised data [25].

EMG signals captured by low-cost Myo armband were used in some recent research [16,30] and were shown to be able to record EMG data. In addition, other works have used Myo armband and its default gesture recognition pattern with a machine learning algorithm to control movements of objects. One of the most recent studies conducted using a Myo armband [33] involved combining the sEMG signal with a Virtual Reality (VR) headset output and implementing Support Vector Machine algorithm to be used for classification. The system was developed to control a robot through eye movements and facial gestures. However, the Myo armband default system in real time has a considerable error rate when recognizing the correct hand pose. Moreover, the number of gestures is limited and the armband contains only five hand gestures.

In the present work, we decided to test raw EMG signals as input of the network. Accordingly, we had to opt for a network which can train with signals. Therefore, we perused recurrent neural network characteristics. As there is little information on using sEMG signals and RNN architectures, we reviewed the results from these architectures with speech signals.

Recurrent neural networks (RNN) have recently proven success in various tasks, especially in machine translation [34,35]. The long short-term memory (LSTM) [36] has shown promising results in a number of natural language processing tasks [34,37], and it was followed by the proposed architecture gated recurrent unit (GRU) [38]. Both architectures have performed well in comparison with ConvNet [39] and on raw speech signal data [40]. Consequently, we decided to utilize sEMG raw signals as an input for our GRU-based network.

## 3. sEMG Dataset Recording and Processing

We studied a number of public datasets, but their sensors did not fit our objective and framework because of the type of sensor and the high number required. Therefore, we decided to create a new dataset for six static hand gestures recorded by Myo armband with the participation of 35 intact individuals.

### 3.1. EMG Sensor Type Discussion

There are two kinds of EMG electrodes: Intramuscular EMG and Surface EMG. Intramuscular EMG has various kinds of recording electrodes; applying needle electrodes is a common method of this category. However, the difficulty of placing them on the arm and connecting them with the muscles correctly make this an arduous task and generates pain and risks for users.

Surface EMG category comprises gel-based and dry electrodes. To be able to use gel-based sEMG, a preparation process is needed before placing the electrodes. The skin must be cleaned and the user’s arm shaved. A conductor gel must then be applied to receive the ideal captured data from electrodes. As the preparation step is lengthy and intricate, this type of sEMG is not an appropriate option to surmount an amputee’s requirements for doing daily routines, which makes them less popular as a long use solution. Although applying dry electrodes reduces preparation time, they still present certain limitations and, with higher impedance, are less accurate compared to gel-based ones [41,42]. Due to the easy access and facilities of handling dry sEMG, in this research, we chose the Myo armband, which has eight dry sEMGs.

### 3.2. Recording Hardware

**Figure 2:**
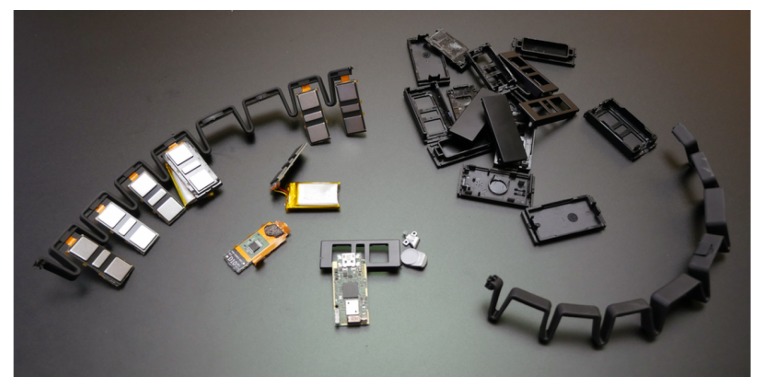
Myo armband tear-down [46].

In recent years, Thalmic Labs(Kitchener, ON, Canada) released a commercial device, the Myo (https://www.myo.com/) armband (the device is available for public unrestricted purchasing), gesture control device composed of a low-consumption ARM Cortex-M4 120 MHZ microprocessor (ArmDeveloper, Cambridge, United Kingdom), 8 dry sEMG and inertial measurement unit (IMU) with a low-sampling rate (200 Hz) (see Figure 2). It provides two types of output data: spatial data and gestural data. The spatial data records the orientation and movements of the user’s arm by 9-axis IMU. Gestural data is recorded by 8 sEMG and gives the information on electrical activity produced by skeletal muscles (This research did not focus on spatial data and just uses EMG data). The use of the Myo armband device has been studied in multiple studies using deep learning architectures [30,43,44,45].

### 3.3. Recording and Labeling Data

In this project, 35 healthy and able-bodied subjects cooperated in the recording of data and labeling hand gestures. The dataset was collected from intact persons of different genders, ages and physical conditions (height and weight).

**Figure 3:**
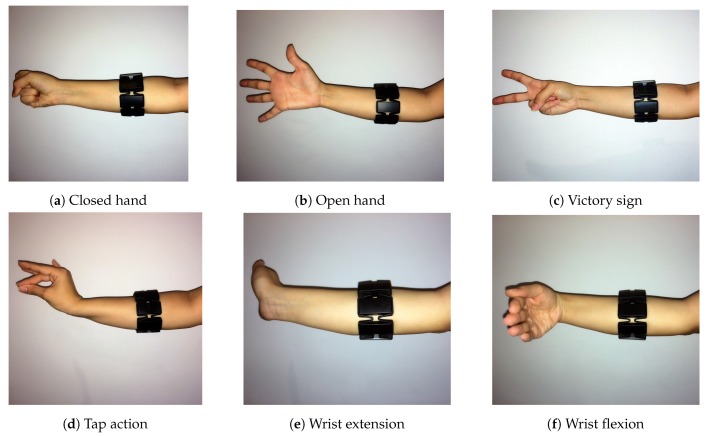
Hand gestures.

Six dissimilar hand gestures (open hand, closed hand, wrist extension, wrist flexion, tap and victory sign) were chosen to train and test the system (See Figure 3). The Myo armband was placed at the height of the Radio-humeral joint, being calibrated for right forearm and right hand gestures. As Myo seemed stable to the external factors [30], special treatment was not needed to begin the data acquisition. However, many factors can affect the sEMG signals [47,48] and should be considered.

**Figure 4:**
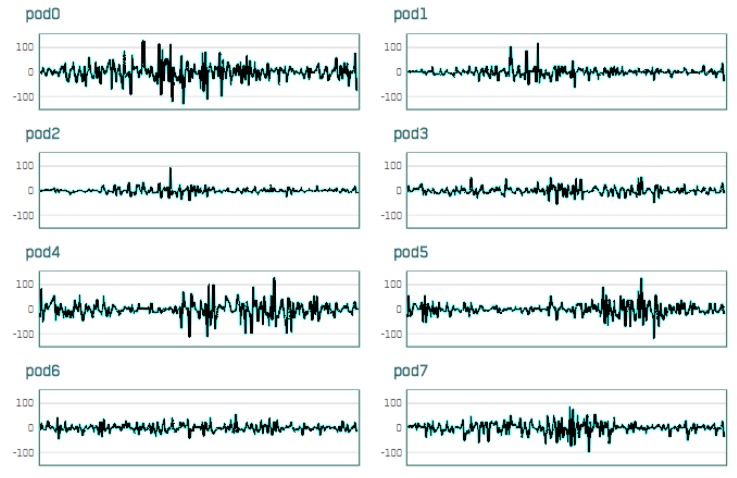
Raw signals (eight) captured with by Myo armband device.

Before the data recording process started, all subjects were given an oral explanation about the experiments and the risks, and were asked to complete an information form. For the sake of completeness and system training, during the data capturing process, one condition was determined. All the subjects were asked to maintain the requested hand gesture, move their arms in various directions for 10 s for each gesture to have more than one Degree Of Freedom (DOF), and avoid complete bending of the forearm at the elbow joint to prevent unwanted effects of brachialis muscle on sEMG signals. Between each gesture recording process, subjects were given a few minutes to rest their arm. Data from Myo was transmitted via Bluetooth at slightly less than 200 Hz in eight channels for each sample (See Figure 4). From the 35 healthy subjects, we acquired approximately 41,000 samples. Twenty-eight subjects were taken for the data training and validation (70% of sample for data training and 30% of sample for data validation), and seven people for test examination whose information was not received by the system during the training process.

## 4. System Description

**Figure 5:**
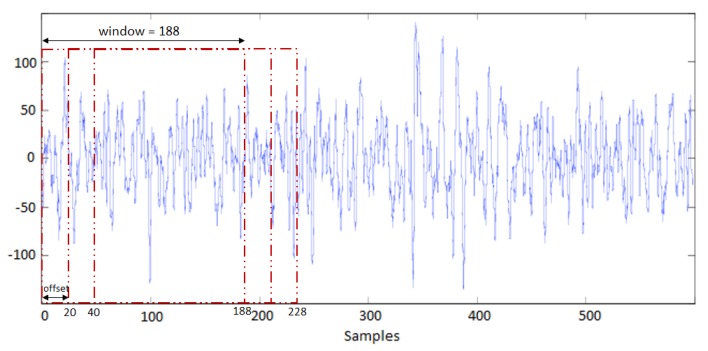
Window method implemented on input data.

In this work, we used a GRU network to process raw sEMG signals. No pre-processing process on the sEMG signal recorded by Myo was necessary. By default, the recorded signal was normalized in the range of −128 to 127 and other methods of normalization were implemented such as normalizing per channel and normalizing between −1 and 1. Nevertheless, the best accuracy was obtained by using the default output of the armband as it is naturally zero-centered. To achieve a better result, we used the window method. Following this method, multiple recent time steps can be used to make a prediction for the next time step. We tried several quantities for the window method and considered 188 as the look-back per sample which had the best outcome with 20 as offset in each window. The look-back number is dependent on the armband frequency that generates almost 188 samples per second and a second is the estimated time that a subject needs to make the gesture correctly in static hand gestures. Figure 5 corresponds to one of the eight channels that are used.

Our neural network contains GRU units, dropout and a fully connected layer. The number of GRU units was tested in several training processes regarding the highest accuracy for the data test.

### Gated Recurrent Unit Network

In this work, the implemented GRU type is the default one based on [38]; GRU is a variant of LSTM, with fewer parameters and faster processing time. It does not use the memory unit to control the data and its reset gate was applied to the hidden state; it can utilize hidden states freely without observation.

**Figure 6:**
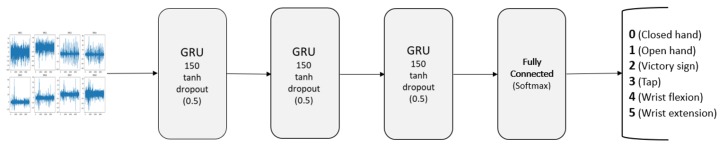
Proposed neural network architecture for hand gesture recognition.

Our proposed classifier follows a recurrent approach. These kinds of methods work with sequences of data to extract temporal features that enable classification cues. Our proposed network features three sequential GRU layers followed by a fully connected layer. Each GRU layer is composed of 150 units with a hyperbolic tangent (tanh) activation function. Each GRU layer is connected to a dropout layer. These layers inhibit random neuron activations with a 0.5 probability. The effect caused by this is twofold: on one hand, the network learns to deal with slightly altered or missing input data. On the other hand, with each iteration, the network is actually training a different architecture as some connections are inhibited. Thus, this layer helps fight overfitting and benefits the generalization capabilities of the final model. Finally, our proposal features a last fully connected (FC) layer with six neurons matching the number of classes of our problem. This final layer is a traditional Multi Layer Perceptron in which neurons have complete linkage to those in the GRU layer’s output. The output from GRU carries important features of the sEMG signals and the fully connected layer uses these features to classify the input signal into existing classes based on the training dataset. This layer uses a softmax activation function that helps control the actions of extreme values without omitting them from the dataset. The described architecture is shown in Figure 6.

The parameters of the architecture such as number of layers, number of neurons per layer, activation functions, normalization layers and dropout rates were empirically chosen.

Finally, it is worth noting that the input data of the network features a batch_size×time_steps×channels shape. The batch_size is the number of samples in one iteration of the training algorithm. In our case, there were 500 samples in each batch. As mentioned, we considered 188 EMG readings per samples, thus time_steps corresponds to the second dimension. The channel dimension corresponds to the readings of the eight different surface sensors of the Myo armband.

For the loss function, we used the categorical-crossentropy function, which means we received a vector with six dimensions as output, which are all zero except one in the prediction index of gesture classes.

## 5. Experiments and Results

**Figure 7:**
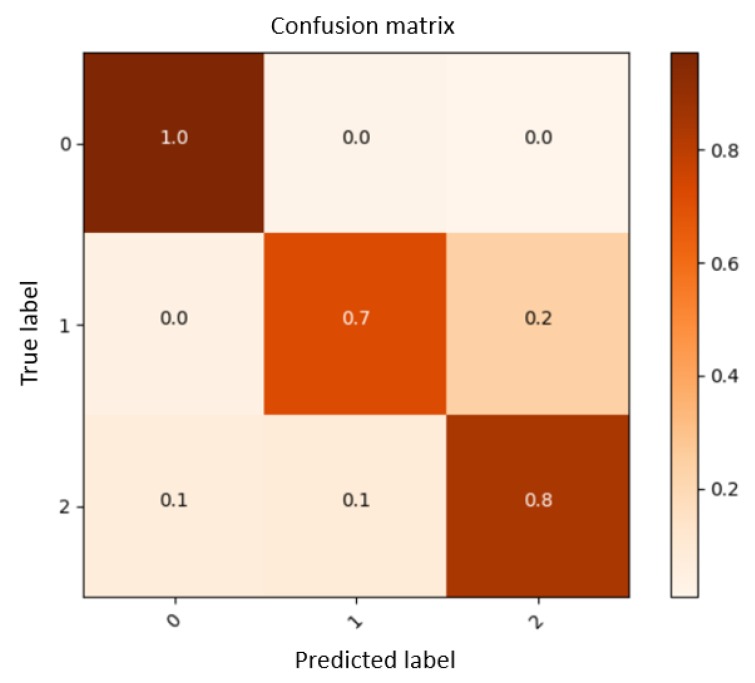
Confusion matrix for three gestures.

Initially, in order to choose the most efficient architecture for the neural network, we divided our experiment into two phases. First, we recorded three basic hand gestures with almost 6000 samples from 10 subjects via the armband (closed hand, open hand and victory sign), and examined the output to check the competence of the armband and the GRU network (see Figure 7).

Based on the preliminary experiments and in order to expand the dataset, we selected 10 dissimilar hand gestures and trained the network, but the Myo armband and its eight sensors were not sufficient to recognize the difference in all the gestures, with test results being around 40%. According to the evaluation procedure described in previous works [8,30], a Myo armband alone is not enough for more than 6–7 gestures [16] and, for more gestures, it should be combined with at least two separate sEMGs placed on flexor digitorum superficialis and extensor digitorum active spots.

**Figure 8:**
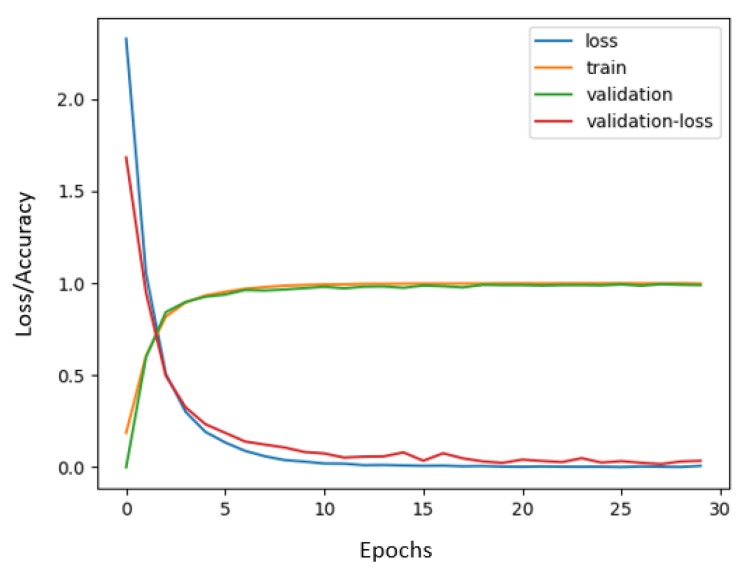
Accuracy and loss graph during the training process.

For more realistic results, we divided the dataset into five groups. Subsequently, we implemented the leave-one-out Cross-Validation technique (35 healthy subjects were divided into groups of seven people) and the training process was conducted via GeForce GTX 1080Ti GPU (Santa Clara, CA, USA). As one group was left out per test, the data for the rest of the subjects was shuffled and divided into 70% for training data and 30% for validation data. The system did not see the validation split during the training process and validation data was from the same subjects as training data but with different samples. In all groups, training accuracy was between 99.40–99.97% as can be seen in Figure 8, which means that the system was learning and could distinguish all gestures. Validation accuracy follows training accuracy during the process with an insignificant difference.

The Cross-Validation experiment includes the training process repeated five times. Each time, four groups were considered as data training, one group as data testing and all five groups were examined as data tests at different times in order to survey the system more accurately. With respect to the Cross-Validation results, the average test accuracy is estimated to be around 77.85%.

It is worth noting at this point that we adopted an early stopping criteria, so the training processes were halted just before an overfitting event or upon stabilization of losses/accuracies. As a result, all experiments were training for about 300 epochs, which were delayed for about 36 h each in the mentioned GeForce GTX 1080Ti GPU. Finally, the optimizer of choice was Adam with a learning rate starting at 0.0001. As concluded in [49], Adam was the best performer, so we adopted it.

**Figure 9:**
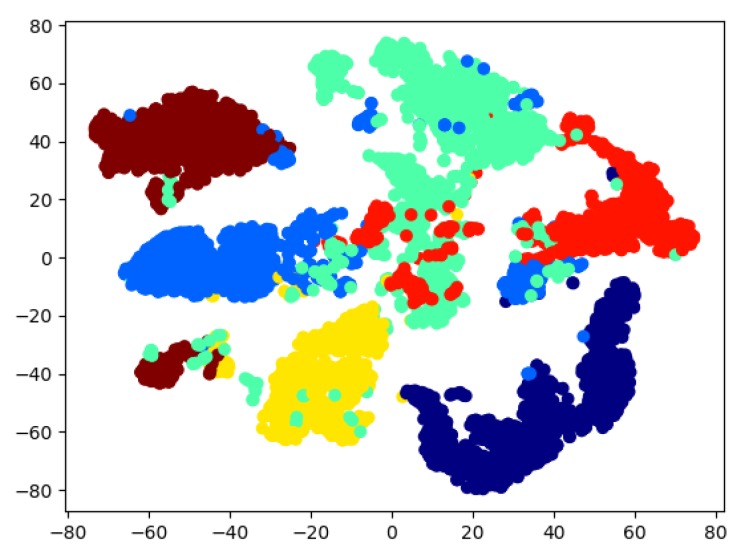
Categorize data by features via T-SNE.

We also implemented a T-distributed Stochastic Neighbor Embedding(T-SNE) algorithm to carry out a dimensionality reduction in 2D for the visualization of our high-dimensional data in Figure 9. In this figure, the training data reduced to two dimensions is plotted. It can be observed that the clusters (different colors indicate different gestures) are separated and, therefore, can be learned by a machine learning method.

In some groups of features, there are errors, which can be seen in the different colors in Figure 9. This means that the system was unable to predict the gestures correctly.

**Figure 10:**
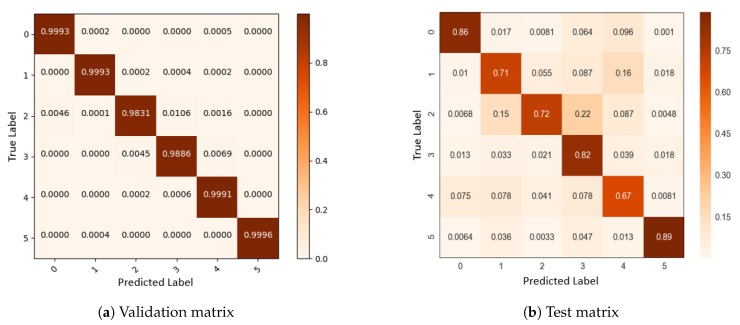
Confusion matrix.

A more accurate estimation of the error percentage can be seen in the confusion matrix (there is a probability of maximum ±0.05 error in each row or column, due to the rounding of numbers). In accordance with the information educed from the confusion matrix in Figure 10, the supposition that GRU architecture is qualified for the gesture recognition system can be proved. Moreover, the confusion matrices demonstrate that the system for subjects who were trained on different samples (validation data) is reasonably accurate. Then, Figure 10b shows a confusion matrix for the test split. It shows that the proposed system is able to successfully classify proposed hand poses even for new subjects.

As the final experiments in this section to test our proposed architecture for gesture recognition, we implemented a live system. The system receives Myo armband information, passes it through the neural network, loads training weights and infers the hand gestures. We ran the live system for five new subjects and found an average accuracy of 80%. According to the results of the live system, there were inconsistencies only between the Victory-sign gesture and the others; for the remaining gestures, we obtained appropriate results. Regarding the runtime, the system is able to provide a prediction in 42 ms in a GeForce GTX 1050 GPU, which means it performs at about 23 frames per second.

**Figure 11:**
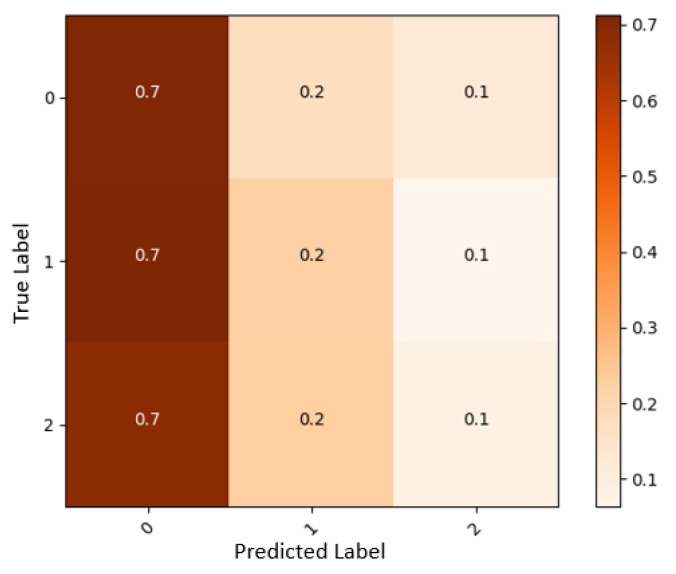
Confusion matrix from a patient with CMT disease.

One of the recorded subjects was suffering from Charcot–Marie–Tooth disease (CMT) (https://www.cmtausa.org/understanding-cmt/what-is-cmt/), although he was healthy and had no movement restrictions. We noticed this patient’s sEMG signals of the patient were totally dissimilar and inconsistent with those of the other subjects. At different stages, this patient was used as a data test so we could compare his results with the other subjects (See Figure 11). As Johnson et al. indicate, patients with CMT have muscle cramps which could affect sEMG signals [50]. Hence, we decided to replace this patient’s data with that of an able-bodied subject in our dataset. This finding suggests there might be probabilities of detecting neuromuscular diseases with this system, which could be studied and developed in future works.

## 6. Conclusions

In this paper, we describe a new approach for live gesture classification with GRU architecture trained by sEMG raw signals acquired from the Myo armband. A new dataset containing approximately 41,000 samples for six dissimilar static hand gestures was created. The proposed GRU classifier was evaluated on different validation sets obtaining an accuracy of 77.85%, to be developed and used for classifying a set of hand gestures performed by a human being. We also studied the correct prediction probability for each gesture and their conflict with other gestures. Moreover, we performed live experiments with multiple new users, verifying that the system was able to distinguish approximately 80% of the trained gestures. In addition, according our experiments’ results, we showed that the GRU network is accurate enough to be used in gesture recognition systems. Future works will focus on developing our dataset, augmenting further hand gestures and testing the system to control a prosthetic hand.
